# Editorial: New experimental and numerical insights on cardiovascular biomechanics through *in-vivo* and *ex-vivo* methods

**DOI:** 10.3389/fphys.2023.1271692

**Published:** 2023-09-08

**Authors:** Emanuele Vignali, Estefania Peña, Miquel Aguirre, Simona Celi

**Affiliations:** ^1^ BioCardioLab, Fondazione Toscana G. Monasterio, Massa, Italy; ^2^ Applied Mechanics and Bioengineering (AMB), Instituto de Investigación en Ingeniería de Aragón, Universidad de Zaragoza, Zaragoza, Spain; ^3^ CIBER de Bioingeniería, Biomateriales y Nanomedicina (CIBER-BBN), Zaragoza, Spain; ^4^ Mines Saint-Etienne, University Jean Monnet, Etablissement Français du Sang, INSERM, U 1059 Sainbiose, Saint-Étienne, France; ^5^ Laboratori de Càlcul Numèric, Universitat Politècnica de Catalunya, Barcelona, Spain; ^6^ International Centre for Numerical Methods in Engineering (CIMNE), Barcelona, Spain

**Keywords:** cardiovascular diseases, tissue biomechanics, experimental tissue characterization, cardiovascular tissues microstructure, clinical imaging

The studies collected in this Research Topic are aimed at addressing different key issues concerning new numerical or experimental approaches for *in-vivo* or *ex-vivo* assessment of the biomechanical behavior of cardiovascular tissues and implants/repair procedures. The gathered works cover different districts of the cardiovascular system.

Three works from the current Research Topic were mainly focused on the analysis of aneurysms affecting the abdominal section of the aorta and on the corresponding repair procedures. Abdominal aorta aneurysms (AAAs) are localized inflations of the abdominal aorta section that may degenerate and lead to abrupt vessel rupture. At the current state, AAAs are managed with constant diameter monitoring and endovascular repair. Emendi et al. proposed the validation of a numerical setup to evaluate the effect of guidewire insertion for endovascular aneurysm repair (EVAR) in terms of mechanical deformations on a patient-specific AAA. Firstly, the group manufactured patient-specific AAA phantoms to simulate *in-vitro* the EVAR guidewire insertion. The phantoms were fabricated in order to also include the presence of intraluminal thrombus. Experimental displacement was recorded through computed tomography (CT) image acquisition. Secondly, the displacements were validated in a finite element (FE) environment by imposing a custom simulation of the guidewire insertion in the AAA phantom. The prediction of guidewire-induced deformations was successfully achieved. For the estimation of strains on AAA cases on the basis of *in-vivo* imaging, the work from Bracco et al. was published in the Research Topic as well. The main objective of this study was to develop a fast and semi-automatic method to post-process dynamic clinical ultrasound sequences to obtain cross-sectional strains on AAAs from B-mode echo images. The mapping procedure was first validated using simulated ultrasound sequences, with a maximum root mean square error of 0.22 mm. The strain estimation was compared with the established finite element approach. Agreement was demonstrated, with mean differences smaller than the image spatial resolution. The study from Karlsson et al. focused instead on the *in-vivo* estimation of wall stress and mechanical properties on AAA cases. The group proposed *in-vivo* measurement of diameters-pressures in a group of 30 cases of AAA. The data consisted of ultrasound echo-tracking acquisitions for AAA diameter and micromanometer tip catheter acquisitions for systemic pressure. The *in-vivo* data were fitted according to a fiber-based model in order to evaluate tissue anisotropy. The population study demonstrated a significant difference in terms of AAA wall stress between male and female cases.

Aneurysms may also affect other different cardiovascular districts. The risks of rupture and sudden patient death are still high, while the diameter-based clinical principle for evaluation remains unsatisfactory. The work from Geronzi et al. aimed to define a new criterion to assess the rupture risk of ascending thoracic aorta aneurysms (ATAAs). The proposed strategy tries to go beyond the current diameter assessment and includes local shape features to be correlated with the ATAA growth rate. The growth rate for a total of 50 cases was monitored with CT or magnetic resonance (MR) image acquisitions. The proposed shape features were correlated with the growth rate. The work demonstrates the potential of shape feature detection combined with risk classification criteria as a planning tool for ATAA follow-up. For aneurysms affecting cerebral arteries, Bisighini et al. proposed a numerical method for braided stent deployment simulation. A fast and accurate framework, based on machine learning and reduced order modelling, was successfully developed to support stent planning in saccular intracranial aneurysms. The method was validated with success, revealing an average validation error between the predicted and high-fidelity results always below 0.15 mm.

Atherosclerosis is a well-known pathology, linked with the formation of lipid plaques on vessel walls. The formation of such plaques represents a serious risk, leading to wall stiffening and blood clot formation. Two contributions focused on atherosclerotic tissues in different cardiovascular districts. The work from Caballero et al. is based on a deep learning approach to predict plaque vulnerability in atherosclerotic coronaries. A set of finite element simulations was used to first train a network. Then, the network was successfully used to estimate the stiffness of patient specific coronaries from IVUS images, with relative errors lower than 3%. Bringing new insights on atherosclerotic plaque assessment, the work from Hanly et al. proposed a novel imaging technique. In particular, phosphotungstic acid (PTA) was used as a new stain in contrast enhanced micro computed tomography (CE*μ*CT) to assess microstructural features and plaque compositions on porcine carotids. CE*μ*CT imaging showed a positive correlation in terms of collagen content for the PTA-marked carotids when compared with histological data. Additionally, the CT data were successfully used to reconstruct the carotid geometry, with different layers of vessel wall and atherosclerotic plaque components.

Valves play a key role in the overall cardiovascular context. Valvular leaflet tissues and their status are linked to the formation of pathologies such as valvular stenoses. A relevant contribution to this Research Topic in terms of aortic valve pathologies was also published. The work from Tuscher et al. aimed at finding a correlation between valve calcification formation and interstitial cell (IC) states at the aortic valve level. To achieve this objective, the group characterized the cells’ basal tonus, starting from diseased human aortic valve tissues embedded in hydrogels. To establish the tonus of the cells, induced displacements and shape modifications were assessed through microscope analysis. The results demonstrated that ICs from calcified regions exhibited less effective activation compared to non-calcified tissues.

The studies collected in this Research Topic showcase developing methods for reaching new insights concerning cardiovascular tissue biomechanics analysis and for finalizing implant/procedure simulations. It was demonstrated that, for overcoming the challenge of understanding and modeling cardiovascular biomechanics, different techniques, both numerical and experimental, can provide significant information and lead to new conclusions.

